# Complete Genome Sequence of the Unclassified Iron-Oxidizing, Chemolithoautotrophic *Burkholderiales* Bacterium GJ-E10, Isolated from an Acidic River

**DOI:** 10.1128/genomeA.01455-14

**Published:** 2015-02-05

**Authors:** Jun Fukushima, Fuyumi Tojo, Ryoki Asano, Yayoi Kobayashi, Yoichiro Shimura, Kunihiro Okano, Naoyuki Miyata

**Affiliations:** a Department of Biotechnology, Akita Prefectural University, Akita, Japan; b Biotechnology Center, Akita Prefectural University, Akita, Japan; c Department of Biological Environment, Akita Prefectural University, Akita, Japan

## Abstract

Burkholderiales bacterium GJ-E10, isolated from the Tamagawa River in Akita Prefecture, Japan, is an unclassified, iron-oxidizing chemolithoautotrophic bacterium. Its single circular genome, consisting of 3,276,549 bp, was sequenced by using three types of next-generation sequencers and the sequences were then confirmed by PCR-based Sanger sequencing.

## GENOME ANNOUNCEMENT

The acidophilic Fe(II)-oxidizing bacteria are ubiquitous in sulfate-rich acidic environments, including acid mine drainage, sulfuric acid soil, and spring water. Biological Fe(II)-oxidation activity is important for controlling the oxidation of Fe(II) to Fe(III) and for mobility in the environment (1). Acidithiobacillus ferrooxidans, a representative Fe(II)-oxidizing proteobacterium, has been used for practical applications in the bioleaching of metals from low-grade sulfide ores (2). Such biometallurgical processes have also been applied to metal recovery from industrial waste such as printed circuit boards (3). Known acidophilic Fe(II)-oxidizing bacteria are phylogenetically diverse, belonging to the classes Nitrospira, *Firmicutes*, and *Actinobacteria*, in addition to *Proteobacteria* (4–6). Given the large number of species, the *Proteobacteria* may be a major group of acidophilic Fe(II)-oxidizers. However, this class includes only a few known species such as Acidithiobacillus ferrivorans, Acidiferrobacter thiooxydans, Thiobacillus prosperus, and Ferrovum myxofaciens, in addition to Acidithiobacillus ferrooxidans (7).

We report the genome sequence of a new betaproteobacterial strain GJ-E10, which was found in an acidophilic Fe(II)-oxidizing enrichment culture from acidic river sediment. It oxidizes Fe(II) and grows autotrophically under moderate acidophilic conditions (pH 2.2 to 3.5 and 25°C to 37°C). This bacterium may be classified into the order *Burkholderiales*, but the family and genus cannot be resolved based on the sequence of the 16S rRNA gene. We temporarily named this strain Burkholderiales bacterium GJ-E10.

The genome sequence of GJ-E10 was assembled using 550,000 reads (total, 1.3 Gb; average read length, 2,364 bp; TaKaRa Co., Shiga, Japan) using PacBio *RS* II (Pacific Biosciences, Menlo Park, CA, USA), 16 Gb of paired-end 100-bp reads using HiSeq (Illumina, Hayward, CA, USA), and 92 Mb of 8-kb mate-pair reads (500 bp) using GS Junior (Roche Diagnostic, Basel, Switzerland). The PacBio sequence reads were assembled by the hierarchical genome assembly process (HGAP) algorithm (8), resulting in 13 contigs. The Illumina HiSeq reads and Roche GS Junior reads were introduced for further assembly using the CLC Genomic Workbench with the finishing module (CLC-GW). From this analysis, a single circular contig containing no gaps was assembled. However, 22 low-coverage sites in the contig were found by CLC-GW; therefore, we performed PCR and Sanger sequencing for further confirmation. Based on the PCR analysis, only a single base was incorrect in the original sequence. The total genome consisted of 3,276,549 bp with 66.3% G+C content. A total of 3,087 protein-coding genes, 43 tRNA genes, and a single rRNA operon were predicted with GLIMMER (9) and the Microbial Genome Annotation Pipeline (MiGAP) (10). Plasmids were not found in this organism. There was an operon for nitrogen fixation that contained *nifHDK* and some accessory molecule genes. The genes for RuBisCO and the Calvin cycle for carbon fixation were found. Investigations of the other genes involved in basic and secondary metabolism are in progress.

### Nucleotide sequence accession number.

The complete GJ-E10 genome sequence has been deposited in DDBJ/EMBL/GenBank under accession number AP014683.
